# Decision-making impairments in epilepsy: behavioral and EEG evidence of event-related potential

**DOI:** 10.3389/fnins.2025.1644485

**Published:** 2025-08-05

**Authors:** Sijie An, Qing Chen, Yuwei Zhou, Xiangyu Gao, Siyu Gong, Minghao An, Yimin Liu, Chengjuan Xie, Yubao Jiang, Kai Wang, Xingui Chen

**Affiliations:** ^1^Department of Neurology, The First Affiliated Hospital of Anhui Medical University, Hefei, China; ^2^First School of Clinical Medicine Anhui Medical University, Hefei, China; ^3^Anhui Province Key Laboratory of Cognition and Neuropsychiatric Disorders, Hefei, China; ^4^Department of Neurology, Yuexi County Hospital, Anqing, China

**Keywords:** epilepsy, decision-making, Iowa gambling task (IGT), event-related potentials (ERP), event-related spectral power

## Abstract

**Objective:**

Decision-making is impaired in patients with epilepsy; however, the changes in decision-making in patients with new-onset and seizure-remission epilepsy remain unclear. The aim of this study was to examine decision-making differences in patients with new-onset and seizure-remission epilepsy, as well as the neurophysiological mechanisms.

**Methods:**

This study included 32 patients with new-onset epilepsy, 31 with seizure-remission epilepsy with generalized tonic–clonic seizures, and 34 matched healthy individuals. Simultaneous electroencephalogram was performed using the Iowa gambling task (IGT). Behavioral performance in the IGT was assessed among the three groups, and P300 and theta oscillations were used as electrophysiological indicators to observe decision-making ability.

**Results:**

Patients with new-onset and seizure-remission epilepsy had significantly lower net scores, lower accounts, and lesser P300 amplitudes and theta oscillation power than did healthy individuals. The percent use of negative feedback was positively correlated with the P300 amplitude.

**Conclusion:**

Impaired decision-making in persons with epilepsy is associated with decreased P300 amplitude and attenuated theta oscillations. Decision-making function was impaired despite clinical seizure-remission.

**Significance:**

This study is the first to compare the behavioral differences in decision-making ability between patients with new-onset and seizure-remission epilepsy. The combination of electroencephalographic features reveals neural mechanisms and improves the understanding of epilepsy decision-making.

## 1 Introduction

Epilepsy is a prevalent neurological disorder characterized by recurring seizures and unpredictable disturbances in brain function, leading to neurobiological, cognitive, psychological, and social ramifications (Fisher et al., 2005). According to the International League Against Epilepsy (ILAE), generalized seizures rapidly involve bilateral hemispheric networks, including absence seizures, myoclonic seizures, and generalized tonic–clonic seizures (GTCS). GTCS is a common form of epileptic seizure (Fisher et al., 2017; Hirsch et al., 2022). Epilepsy affects approximately 70 million people worldwide (Loscher et al., 2020); however, it has received relatively little scholarly attention despite the importance of understanding the mechanisms behind its onset and progression.

Cognitive impairment is frequently observed in people with epilepsy, with an incidence rate of up to 70%. The study indicates that patients with epilepsy are prone to cognitive and behavioral deficits. Factors such as epilepsy type, underlying etiology, age of onset, seizure frequency, and disease course are all considered important influencers of cognition (Operto et al., 2023). A prolonged history of epileptic seizures and repeated episodes of status epilepticus may induce progressive alterations in brain connectivity, which could lead to cognitive deterioration over time (Novak et al., 2022). Individuals with epilepsy may experience attention deficits, and impairments in memory, language, and executive functions associated with frontal brain regions (Hamed, 2009; Li et al., 2020). People with epilepsy exhibit inferior performance compared to healthy counterparts in immediate memory, delayed memory, and learning activities (Tedrus et al., 2020). A high frequency of seizures is associated with adverse outcomes; functional impairments in brain regions are more severe in patients with interictal phases lasting less than 1 year (Vollmar et al., 2011). Although the exact cause of cognitive impairment in epilepsy remains unknown, the effect of epilepsy on cognitive function is both clinically and neuroscientifically significant.

Decision-making is a fundamental human behavior characterized by the maximization of expected rewards (Biernacki et al., 2016). It encompasses computational analysis, risk assessment, and consequence evaluation. Cognition impairments may appear early in epilepsy (Witt and Helmstaedter, 2015). Individuals with inadequately managed epilepsy exhibit impaired decision-making capabilities (Labudda et al., 2009). There is a lack of research on decision-making ability during the remission phase of epilepsy. Investigating decision-making during new-onset and seizure-remission is essential to better understand epilepsy. The Iowa gambling task (IGT) is a widely used tool for assessing ambiguous situations with implicit outcomes and probabilities (Buelow and Suhr, 2009; Bechara et al., 1994). Its probabilities are implicit, and it shows sensitivity in identifying impulse control disorder diseases (Gleichgerrcht et al., 2010).

The P300 component has been widely used in psychological and neurological studies as an indicator of conscious error recognition and response adjustment (Gokcay et al., 2006). Event-related potentials (ERP) research commonly regards the P300 as a marker for decision-making in ambiguous contexts (Zheng et al., 2020). Additionally, neural oscillations, particularly theta oscillations, are essential for various neurophysiological and cognitive functions. Theta signals, especially in the frontal cortex, facilitate communication between the frontal executive and parietal attentional control areas and support higher-order cognitive processes (Rajan et al., 2019). When errors or negative feedback occur, theta oscillation power increases in the middle frontal region (Nurislamova et al., 2019). These findings suggest that P300 and theta oscillations are valuable electrophysiological markers for monitoring alterations in decision-making abilities. Therefore, using these metrics to investigate the electrophysiological mechanisms underlying GTCA-related decision-making impairments is a promising approach.

Although many studies have identified behavioral indicators of decision-making dysfunction in patients with epilepsy, few have explored electrophysiological mechanisms underlying these deficits (Donoghue and Voytek, 2022). In new-onset epilepsy patients with recent frequent seizures, the brain neuronal network may be in a stage of plasticity remodeling mediated by abnormal electrical activity (Xing et al., 2024). Studies have shown that the left thalamic volume in children with new-onset epilepsy can be significantly reduced (Perani et al., 2018), and the functional connectivity abnormality of this structure with the prefrontal cognitive regulatory network may directly lead to decision-making related cognitive impairment. Although seizure control is achieved in remission epilepsy patients, persistent abnormal electrical activity may exist in the brain (Clemens et al., 2013), and long-term disease course combined with neurotoxicity of antiepileptic drugs may lead to neural structure remodeling and metabolic reduction in the prefrontal cortex (Majeed et al., 2022), which can still decrease the regulatory efficiency of the executive function network. Comparing the cognitive characteristics of the two groups clarifies the dynamic impact of epilepsy on brain function at the mechanistic level, while providing a basis for clinical hierarchical management. This holds certain significance for improving the overall prevention and treatment level of epilepsy and ameliorating long-term prognosis of patients.

This study focuses on the assessment of cognitive functions in patients with epilepsy. The IGT was employed to quantitatively analyze decision-making abilities. By including drug-naive new-onset patients (to exclude the interference of antiepileptic drugs on cognition), we systematically compared the differences in decision-making functions among new-onset patients, those in remission, and healthy controls. The study integrated electrophysiological analyses of event-related potentials (P300) and theta wave oscillations in the prefrontal cortex. We hypothesized that remission patients would show slightly better performance than new-onset patients, but when compared with healthy controls, the decision-making ability of both epileptic groups was found to decline. Electrophysiological results showed decreased P300 amplitude and prefrontal theta wave power. This study aims to clarify the behavioral characteristics of decision-making disorders in different stages of epilepsy and reveal their underlying neuroelectrophysiological mechanisms.

## 2 Methods

### 2.1 Participants

All patients were recruited from the outpatient clinic and ward of the Department of Neurology at the First Affiliated Hospital of Anhui Medical University. The inclusion criteria were as follows: (1) age of 18–65 years; (2) Patients demonstrating GTCS during video-EEG monitoring, with clinical manifestations consistent with typical tonic–clonic seizure characteristics, as diagnosed by certified epileptologists according to the International League Against Epilepsy (ILAE) classification criteria (Fisher et al., 2017); and (3) new-onset epilepsy characterized by the initial epileptic seizure diagnosed as epilepsy within 6 months and not previously treated with antiepileptic medication (Hermann et al., 2006). Seizure-remission epilepsy is characterized by a seizure-free period of at least 12 months (Itamura et al., 2023), and no anti-seizure medication adjustments have been made within 1 year. The exclusion criteria were (1) the existence of other organic diseases, (2) a history of alcohol-related disorders or the consumption of any substance affecting the central nervous system, and (3) comorbid anxiety or depression.

The study ultimately included 32 individuals with new-onset diagnosis (18 male individuals) and 31 patients in seizure-remission (20 male individuals). Thirty-four healthy individuals (16 male individuals) were matched for age and sex. 18 patients were on anti-seizure medication monotherapy, while 13 patients were on polytherapy.

No substantial differences were observed among the three groups regarding sex (χ^2^ = 3.871, *p* = 0.424), age (*F* = 2.277, *p* = 0.108), or education (*F* = 2.242, *p* = 0.112), with specific demographic details indicated in Table 1. All individuals underwent standardized neuropsychological assessments to evaluate their cognitive abilities at baseline. The Beijing variant of the Montreal Cognitive Assessment Test was used to evaluate overall cognitive performance (Yu et al., 2012). The Hamilton Anxiety Scale (HAMA) (Price et al., 2011) and the Hamilton Depression Scale (HAMD) (Möller, 2001) were used to exclude participants with comorbid anxiety and depression (exclusion criteria: HAMA > 17, HAMD > 14). The Ethics Committee of Anhui Medical University approved all study protocols (Ethical approval number: 2019H022, date: 2020.12.28). All participants provided informed consent before participating in the study. The experiment is in accordance with the ethical principles of the Declaration of Helsinki.

**Table 1:** Demographic background information of participants and IGT performance.

	New-onset (*n* = 32)	Seizure-remission (*n* = 31)	HC (*n* = 34)	χ^2^/*F*	*p*
	Mean (SD)	Mean (SD)	Mean (SD)		
Age (years)	30.66 (5.80)	31.13 (4.57)	28.59 (4.97)	2.277	0.108
Sex (male/female)	32 (18/14)	31 (20/11)	34 (16/18)	3.871	0.424
Education (years)	13.47 (2.29)	13.68 (1.81)	14.38 (1.33)	2.242	0.112
Disease course (months)	3.22 (1.90)	98.16 (74.87)	NA	/	/
HAMA (score)	1.38 (1.16)	0.87 (0.99)	1.35 (1.35)	1.843	0.164
HAMD (score)	0.91 (1.03)	0.87 (1.15)	1.00 (0.20)	0.116	0.890
MoCA	27.31 (2.32)	27.48 (1.48)	28.05 (1.18)	1.711	0.186
IGT total score	54.06 (65.19)	64.00 (65.04)	102.88 (66.94)	5.107	0.008*
IGT money account	303.13 (1020.51)	367.74 (1016.82)	1029.41 (988.73)	5.271	0.007*
The percent use of negative feedback	0.60 (0.16)	0.67 (0.15)	0.70 (0.16)	3.085	0.049*

Values represent mean (SD). NA, not applicable; HAMA, Hamilton Anxiety Rating Scale; HAMD, Hamilton Depression Rating Scale; MoCA, Montreal Cognitive Assessment Test; IGT, Iowa gambling task; HC, healthy control; SD, standard deviation.

### 2.2 IGT

**Figure 1:**
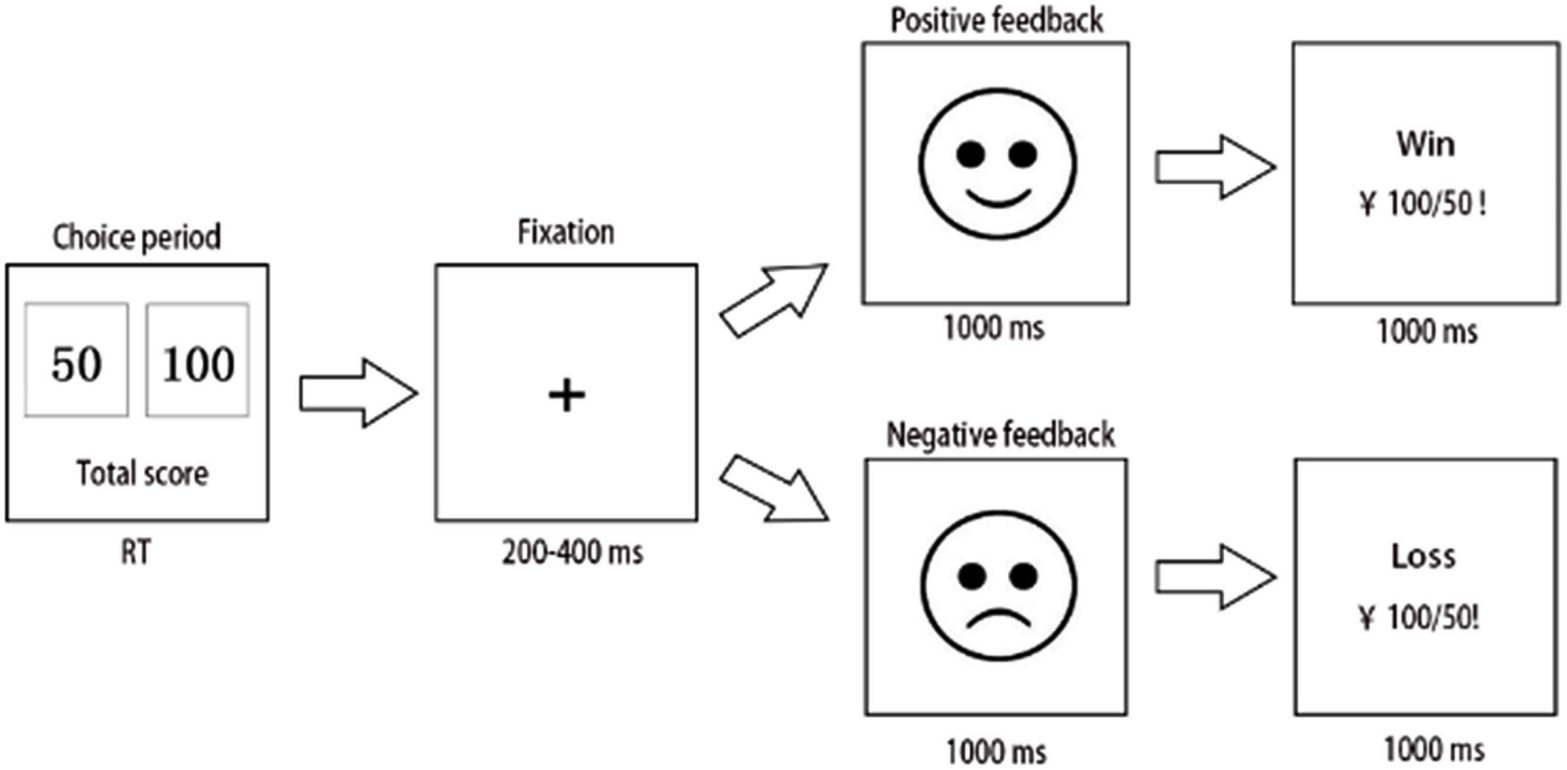
Flowchart of the Iowa gambling task (IGT), with the key F representing 50 and key J representing 100. After each selection, the screen exhibited 200–400 ms of fixation, succeeded by feedback in the form of a cartoon face, which persisted for 1,000 ms until a letter appeared on the computer screen, alerting the participant of the outcome of the choice. The experiment concluded after 300 trials.

A computerized variant of the IGT was used in this experiment. Participants were notified that they would engage in a gambling game, with a selection of two decks of cards displayed on the computer screen, allowing the user to opt for either a “small” (50 RMB) or a “large” (100 RMB) bet. The win/loss sequence for each betting type is randomized, exhibiting a win/loss probability of 0.6/0.4 for a 50-point wager and 0.4/0.6 for a 100-point wager. The most effective technique is to select the “small,” low-risk wager type to optimize the end outcome. The initial investment was 1,000 RMB. After each option, a 200–400 ms pause ensued, followed by feedback, in the form of a cartoon face. The cartoon-based visage endured for 1,000 ms. Subsequently, a message with text and numbers appeared on the computer screen, notifying the participants of the results of their selection. They were instructed to maximize their monetary gain (Figure 1). Furthermore, “high-risk loss” was delineated as when a member selected a high-risk choice and incurred a loss. A “low-risk win” was defined as a scenario in which the player selects the low-risk option and achieves a profit. The percent use of negative feedback was calculated by dividing the number of times a participant transitioned to a lower risk after selecting a high-risk loss by the number of times the participant accepted a high-risk loss. The activity spanned approximately 15 min and had 300 trials. All patients were compensated based on a fixed rate of cash at the end of the experiment according to the final account.

### 2.3 EEG data recording and analysis

EEG recordings were performed in a tranquil and comfortable setting, with the participants encouraged to cleanse their scalps before the experiment and remain awake and attentive. Electrode scalp elastic caps were positioned according to the international 10–20 system, and EEG was performed using Neuro Scan equipment (Neuro Scan, Sterling, VA, United States). The electrode on the forehead was the grounding electrode, and all EEG channels were referenced to the left mastoid (Choi et al., 2022). A vertical electrooculogram was placed 1 cm above the middle of the left eyebrow and 1 cm below the left pupil, whereas a horizontal electrooculogram was placed 1 cm lateral to the two ocular lobes to assess the open and closed states of the eyes. All electrode impedances were maintained below 5 KΩ. EEG activity was enhanced by band-pass filtering from 0.01 to 100 Hz and continuously captured at 1,000 Hz per channel.

The EEG data were processed and analyzed using MATLAB scripts within the EEGLAB framework (Edwards et al., 2013). The average values of the right and left mastoids were re-referenced and down-sampled to 500 Hz. Eye movements, electromyographic activity, and other non-artifactual components were eliminated by independent component analysis using the EEGLAB toolbox. Midline loci (FZ, CZ, and CPZ) exhibit greater P300 amplitudes (Helfrich and Knight, 2019). A waveform period of [−0.2, 1] was selected to generate group-level ERP plots, and the mean P300 amplitude was calculated over a 350–450 ms time window for statistical analysis. Theta oscillations were analyzed using the STUDY panel within the EEGLAB framework. A frequency range of 4–6 Hz and a temporal window of 350–450 ms were designated as the criteria for theta oscillations based on prior research and current time-frequency representations. The mean power (dB) within this time frame was extracted for statistical evaluation and subsequently illustrated (Soltani Zangbar et al., 2020).

### 2.4 Statistical analyses

All behavioral and electrophysiological analyses were conducted using SPSS software (version 17.0; SPSS Inc., Chicago, IL, United States). Comparisons across the sexes were conducted using the chi-square test. The aggregate of the net scores from the five block groups constituted the overall net scores, whereas the final account at the conclusion of the game represented the IGT account. A one-way analysis of variance (ANOVA) was used to examine the disparities among the three groups in the IGT total net scores, IGT accounts, and the percent use of negative feedback. The behavioral experiments were segmented into five blocks, with net scores calculated as the difference between the number of favorable and unfavorable choices in each block. The block served as a within-patient factor, whereas the group functioned as a between-patient factor. The decision-making performance of the three groups was assessed using a 3 (group) × 5 (block) repeated-measures ANOVA. Multiple comparisons among the three groups were conducted using Bonferroni adjustment. The mean amplitude of the event-related potential P300 and the mean power of theta oscillations were examined using repeated-measures ANOVA, with the type of feedback (win/loss), intensity of risk (50 for low risk and 100 for high risk), and electrodes (Fz, FCZ, CZ, and CPZ) as within-patient factors and the group as a between-patient factor. Pearson’s correlation coefficients were calculated to examine the strength of the associations between P300 amplitude and the percent use of negative feedback. A *p*-value of < 0.05 was considered significant.

## 3 Results

### 3.1 Demographics and behavioral performance

**Figure 2:**
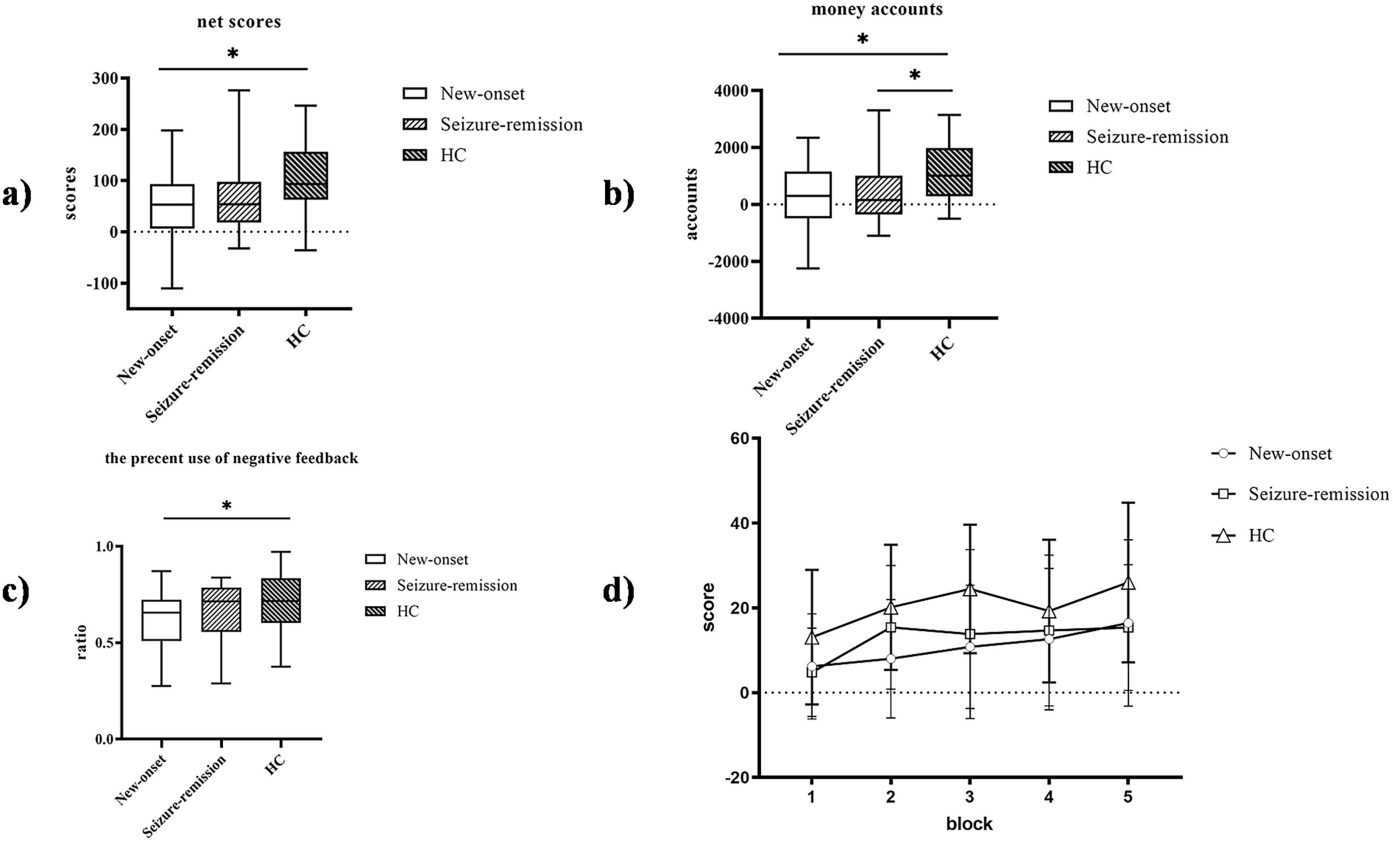
Performance of the three groups on the Iowa gambling task (IGT). The IGT net scores were compared among the three groups. **(a)** The IGT net scores. **(b)** The money account. **(c)** The percent use of negative feedback. **(d)** Comparison of the net scores of the three patient groups in the five blocks. Bars indicate the standard error of the mean. * *p* < 0.05. HC, healthy control.

The IGT net scores [*F* = 5.107, *p* = 0.008] exhibited significant variation among the three groups (Figure 2). The IGT net scores of the healthy individuals surpassed those of the epilepsy group. Furthermore, multiple comparisons revealed significant differences in the IGT net scores between the new-onset epilepsy and healthy groups (*p* = 0.003), as well as between the seizure-remission and healthy groups (*p* = 0.019). No significant difference was observed in the IGT net scores between the new-onset epilepsy and seizure-remission groups (*p* = 0.550). The money account of the IGT exhibited significant variation among the three groups, being higher in healthy individuals than in the two epilepsy groups [*F* = 5.271, *p* = 0.007]. Multiple comparisons revealed significant differences between the new-onset epilepsy and healthy groups (*p* = 0.004), as well as between the seizure-remission and healthy groups (*p* = 0.010). No significant difference was observed between the new-onset epilepsy and seizure-remission groups (*p* = 0.800). The percent use of negative feedback [*F* = 3.085, *p* = 0.05] differed significantly among the three groups, and multiple comparisons of the difference between new-onset epilepsy and healthy individuals were significant (*p* = 0.017). No significant difference was observed between the new-onset epilepsy and seizure-remission groups (*p* = 0.106), nor between the healthy and seizure-remission groups (*p* = 0.447).

A significant within-patient effect of block [*F* = 15.614, *p* = 0.001] and a significant between-patient effect of group [*F* = 5.107, *p* = 0.008] was observed, suggesting a dynamic process in IGT performance. Paired comparisons showed no significant difference between the new-onset and remission groups (*p* = 1.000), a significant difference between the new-onset and healthy groups (*p* = 0.010), and between the remission and healthy groups (*p* = 0.058). Additionally, there was a significant interaction between block and group [*F* = 2.125, *p* = 0.036], indicating that decision-making strategies varied among different groups. Analysis of the simple effects of group and block showed a significant difference in the net scores between patients with new-onset epilepsy and healthy individuals (*p* = 0.01) and a borderline significant difference between patients in seizure-remission and healthy individuals (*p* = 0.058). In blocks 1 and 3, a significant difference was observed in the net scores between the healthy and seizure-remission groups (*p* = 0.039 and *p* = 0.034, respectively), and in block 2, a significant difference was observed between the healthy and new-onset epilepsy groups (*p* = 0.003).

### 3.2 P300 amplitude

**Figure 3:**
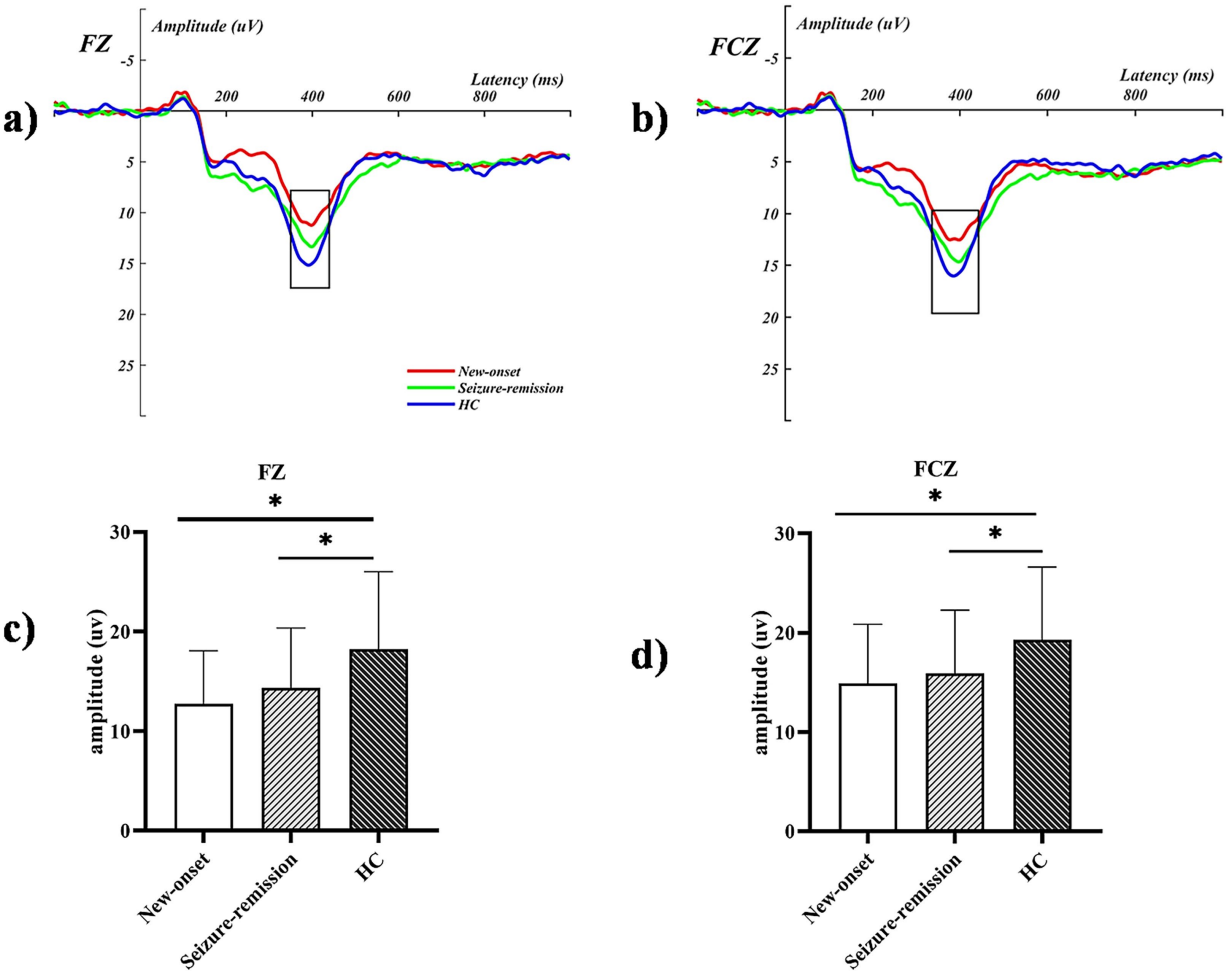
The event-related potential graphs for the electrodes **(a)** FZ and **(b)** FCZ in the loss-100 condition illustrate significant amplitude increases within the defined time frames marked by the black boxes. The P300 mean amplitude analysis for the corresponding extracted time windows is presented in **(c,d)**, with bars representing the standard error of the mean. * *p* < 0.05. HC, healthy control.

Significant effects were observed for the intensity [*F* = 87.979, *p* = 0.001], feedback type [*F* = 9.645, *p* = 0.003], and electrode [*F* = 38.221, *p* = 0.001]. Furthermore, significant interactions were observed between feedback type and group [*F* = 3.737, *p* = 0.027], intensity and group [*F* = 8.184, *p* = 0.001], and electrode and group [*F* = 2.162, *p* = 0.048]. Univariate tests revealed that an intensity of 100 elicited a significantly higher wave amplitude, compared with an intensity of 50. A significant difference was observed among the three groups in the loss-100 condition (*F* = 3.476, *p* = 0.035), and further analysis revealed a significant difference between the healthy and new-onset epilepsy groups (*p* = 0.04). No significant difference was observed between the new-onset epilepsy and remission groups (*p* = 1.000), nor between the remission and healthy groups (*p* = 0.186). Wave amplitudes differed significantly between the healthy and new-onset epilepsy groups at the FCZ in the loss-100 condition (*p* = 0.024). No significant difference was observed between the new-onset epilepsy and remission groups (*p* = 1.000), nor between the remission and healthy groups (*p* = 0.123). A notable disparity was observed between the new-onset epilepsy and healthy groups (*p* = 0.004), as well as between the seizure-remission and healthy groups (*p* = 0.05). By contrast, no significant difference was found between the new-onset epilepsy and seizure-remission groups (*p* = 1.000) at the FZ in the loss-100 condition. Table 2 presents the analysis of the simple effects of feedback type, intensity, electrode, and group. The average ERP waveforms of the three groups at FCZ and CZ for the loss-100 conditions are shown in Figure 3.

**Table 2:** Repeated measures ANOVA for P300 amplitude (μV) across the three groups.

	New-onset (*n* = 32)	Seizure-remission (*n* = 31)	HC (*n* = 34)	*F*	*p*
	Mean (SD)	Mean (SD)	Mean (SD)		
FZ50	11.16 (5.47)	14.07 (6.53)	13.47 (7.17)	1.817	0.168
FZ51	9.10 (4.44)	10.94 (4.60)	10.76 (5.89)	1.298	0.278
FZ100	13.00 (5.20)	14.34 (6.03)	18.25 (7.77)	5.909	0.004*
FZ101	12.57 (5.46)	13.29 (5.28)	15.03 (8.52)	1.202	0.305
FCZ50	13.01 (6.22)	15.69 (7.00)	14.63 (7.16)	1.234	0.296
FCZ51	10.88 (5.53)	12.58 (4.95)	11.90 (5.93)	0.765	0.468
FCZ100	14.90 (5.96)	15.91 (6.38)	19.30 (7.32)	4.061	0.020*
FCZ101	14.78 (6.68)	15.29 (5.25)	16.13 (8.18)	0.328	0.721
CZ50	13.12 (6.29)	15.90 (7.25)	14.16 (6.30)	1.419	0.247
CZ51	11.95 (5.70)	13.14 (5.05)	12.06 (5.42)	0.467	0.629
CZ100	15.01 (5.89)	15.92 (6.45)	18.45 (6.47)	2.672	0.074
CZ101	15.59 (6.81)	15.82 (5.12)	16.43 (7.48)	0.146	0.864
CPZ50	12.81 (6.30)	15.21 (7.10)	13.20 (6.09)	1.231	0.297
CPZ51	12.40 (5.59)	13.11 (4.65)	11.66 (5.21)	0.640	0.530
CPZ100	14.88 (5.91)	15.36 (6.45)	17.09 (6.47)	1.144	0.323
CPZ101	15.80 (6.47)	15.65 (4.80)	15.46 (6.91)	0.025	0.976

FZ50, FCZ50, CZ50, CPZ50: loss-50 condition; FZ51, FCZ51, CZ51, CPZ51: win-50 condition; FZ100, FCZ100, CZ100, CPZ100: loss-100 condition; FZ101, FCZ101, CZ100, CPZ100: win-100 condition, * *p* < 0.05. ANOVA, analysis of variance; SD, standard deviation.

### 3.3 Theta oscillations

**Figure 4:**
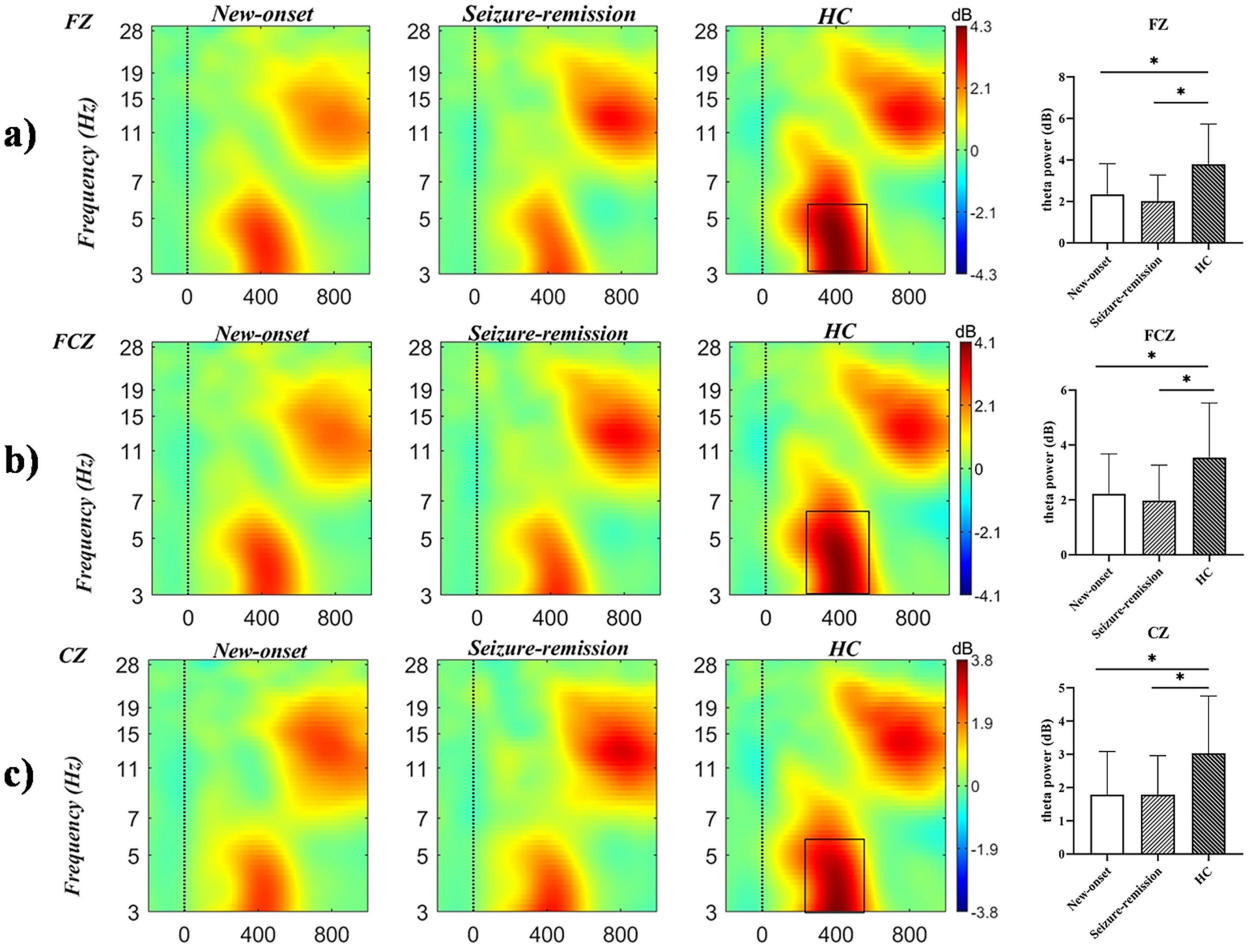
Time-frequency representations of theta oscillations at electrodes **(a)** FZ, **(b)** FCZ, and **(c)** CZ were generated using MATLAB, with the black box delineating the time-frequency region of interest where power exhibits a significant increase. Differences in theta oscillatory activity among the three electrodes are illustrated (bars represent the standard error of the mean; * *p* < 0.05). HC, healthy control.

A significant effect was observed for intensity [*F* = 15.306, *p* = 0.001] but not for the electrode [*F* = 1.207, *p* = 0.312] or feedback type [*F* = 0.059, *p* = 0.809]. Significant between-patient group effects [*F* = 4.574, *p* = 0.013] were observed, and *post hoc* comparisons revealed significant differences between the healthy and new-onset epilepsy groups (*p* = 0.026) and between the healthy and seizure-remission groups (*p* = 0.041). No significant difference was found between the new-onset epilepsy and seizure-remission groups (*p* = 1.000). A notable interaction was observed between feedback type and intensity (*F* = 69.810, *p* = 0.001) and among feedback type, intensity, and group (*F* = 3.959, *p* = 0.022). Univariate analyses revealed significant differences among the three groups regarding the type of feedback for loss [*F* = 6.645, *p* = 0.002]. Significant differences were found in the comparisons of the healthy group with the new-onset group (*p* = 0.006) and with the seizure-remission group (*p* = 0.007). No significant difference was observed between the new-onset epilepsy group and the seizure-remission group (*p* = 1.000). There were significant difference among the three groups in the 100 intensity condition [*F* = 4.609, *p* = 0.012]. Further analysis revealed significant differences between the healthy and seizure-remission groups, as well as between the healthy individuals and new-onset epilepsy groups (*p* = 0.032 and *p* = 0.031, respectively). No significant difference was observed between the new-onset epilepsy and seizure-remission groups (*p* = 1.000). Additionally, theta oscillation induced at the intensity of 100 was higher than at the intensity of 50. *Post hoc* 2 × 2 comparison showed that, at the FZ, FCZ, CZ, and CPZ electrodes, the healthy group differed significantly from both the seizure-remission and new-onset epilepsy groups (*p* < 0.05). However, no significant difference was observed between the new-onset epilepsy and seizure-remission groups (*p* > 0.05). The results of the specific analyses are presented in Table 3. The average theta oscillations power of the three groups at the FZ, FCZ, and CZ electrodes under the loss-100 condition is shown in Figure 4.

**Table 3:** Repeated measures ANOVA for theta oscillations (dB) across the three groups.

	New-onset (*n* = 32)	Seizure-remission (*n* = 31)	HC (*n* = 34)	*F*	*p*
	Mean (SD)	Mean (SD)	Mean (SD)		
FZ50	2.11 (1.43)	1.98 (1.33)	2.94 (1.97)	3.460	0.035
FZ51	0.69 (0.93)	0.65 (1.11)	1.30 (1.42)	3.156	0.047
FZ100	2.33 (1.49)	2.01 (1.27)	3.79 (1.95)	11.566	0.001*
FZ101	1.05 (1.21)	0.97 (1.52)	1.38 (1.57)	0.730	0.484
FCZ50	1.95 (1.52)	1.86 (1.32)	2.81 (1.99)	3.366	0.039
FCZ51	0.65 (1.07)	0.74 (1.22)	1.31 (1.48)	2.613	0.079
FCZ100	2.22 (1.46)	1.97 (1.30)	3.55 (1.98)	9.012	0.001*
FCZ101	1.04 (1.20)	1.11 (1.66)	1.43 (1.56)	0.657	0.521
CZ50	1.46 (1.51)	1.66 (1.31)	2.44 (1.71)	3.811	0.026*
CZ51	0.85 (1.27)	1.12 (1.42)	1.55 (1.55)	2.043	0.135
CZ100	1.79 (1.31)	1.78 (1.18)	3.04 (1.73)	8.423	0.001*
CZ101	1.28 (1.35)	1.52 (2.19)	1.85 (1.76)	0.842	0.434
CPZ50	1.15 (1.26)	1.38 (1.37)	2.09 (1.61)	3.925	0.023*
CPZ51	1.10 (1.33)	1.35 (1.48)	1.59 (1.55)	0.906	0.408
CPZ100	1.51 (1.16)	1.57 (1.14)	2.58 (1.77)	6.083	0.003
CPZ101	1.50 (1.49)	1.69 (2.31)	1.98 (1.87)	0.538	0.586

FZ50, FCZ50, CZ50, CPZ50: loss-50 condition; FZ51, FCZ51, CZ51, CPZ51: win-50 condition; FZ100, FCZ100, CZ100, CPZ100: loss-100 condition; FZ101, FCZ101, CZ100, CPZ100: win-100 condition, * *p* < 0.05. ANOVA, analysis of variance; SD, standard deviation.

### 3.4 Correlation analysis

**Figure 5:**
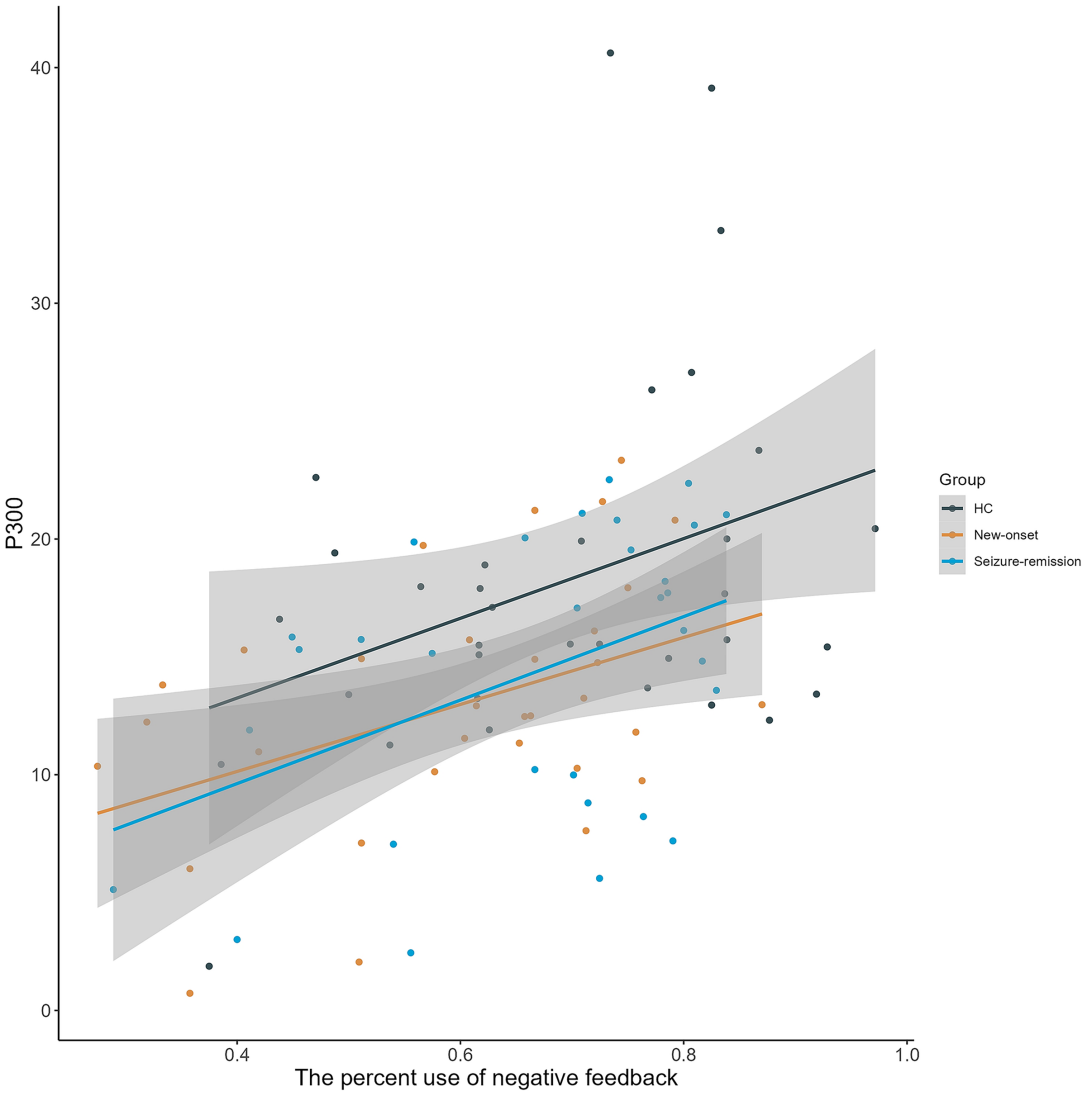
Scatterplot illustrating the relationship between the percentage use of negative feedback and P300 amplitude elicited by a high chance of loss at FZ across three groups, demonstrating a substantial positive association (grey-shaded regions represent 95% confidence intervals). HC, healthy control.

The relationship between behavioral markers of decision-making and electrophysiological indicators was investigated in all individuals (Figure 5). In all the three groups, there was a significant correlation between the percentage use of negative feedback and the P300 amplitude at the FZ electrode in the loss-100 condition (new-onset group: r = 0.371, *p* = 0.031; seizure-remission group: r = 0.439, *p* = 0.013; healthy group: *F* = 0.350, *p* = 0.042).

## 4 Discussion

This study examined disparities in decision-making ability and electrophysiological mechanisms among individuals with new-onset GTCS, those in seizure-remission and healthy controls under ambiguous risk conditions. The study results indicated inferior decision-making in patients with new-onset epilepsy and those in seizure-remission compared with their healthy counterparts, and the decline in decision-making ability may be associated with reduced P300 amplitude and theta oscillations. This study also observed that patients with epilepsy had lower P300 amplitude and theta oscillation power, and the percent use of negative feedback loss than did the healthy group. Furthermore, the percent use of negative feedback demonstrated a significant positive correlation with P300 amplitude. These results suggest that patients continue to experience lingering deficits in their decision-making capacities despite clinical seizure-remission.

Consistent with prior studies on juvenile myoclonic epilepsy (JME), JME patients exhibiting deficient executive function are more prone to engage in risky decision-making compared to healthy controls (Zamarian et al., 2013). Research has revealed impaired decision-making capabilities in persons with cryptogenic frontal lobe epilepsy and temporal lobe epilepsy. In the present study, people with GTCS performed worse, compared with healthy individuals, in terms of the IGT net scores, IGT accounts, and percent use of negative feedback (Delazer et al., 2010; Yamano et al., 2011). In the new-onset epilepsy group, performance initially increased in the second block but then leveled off, whereas healthy individuals displayed an overall upward trend. Across all blocks, the people with epilepsy group performed worse than the healthy group did, showing challenges in developing and maintaining favorable long-term decision-making strategies. Emotional regulation and decision-making processes appear to be affected when patients face risky situations. The orbital frontal cortical (OFC), anterior cingulate, and ventromedial prefrontal cortex (VMPFC), plays a crucial role in decision-making behavior. Patients with inadequately managed seizures often show poorer performance on the IGT and other executive function assessments, likely owing to cortical dysfunction from prolonged seizures and reduced connectivity in neural networks (Kanner and Bicchi, 2022; Brand et al., 2007). The OFC is involved in decision-making, risk aversion, and relearning (Balewski et al., 2023) and conveys external information to the hypothalamus and amygdala through the VMPFC. The thalamus activates subnetworks in the frontal cortex that influence various aspects of decision-making (Yang et al., 2022). Imaging studies have shown that changes in subcortical areas linked to epilepsy and weakened connectivity in the frontal-thalamic network may impair decision-making (Zhang et al., 2011). Reduced connectivity between the OFC and VMPFC may also impair contingency relearning, as observed in patients who struggle to adjust their strategies after failure, contributing to their compromised decision-making ability. From a neurobiological perspective, the weakened regulation of the prefrontal cortex over the limbic system makes individuals more susceptible to emotional drives, leading to impulsive behaviors. This reduction in neural regulation directly manifests as a decline in decision-making ability in behavioral terms (Jones et al., 2021). The low percent use of negative feedback in the epilepsy group indicates slower strategy adjustment or difficulty in effectively using negative feedback to optimize decision-making, which aligns with studies on decision-making impairment in addiction models suggesting an inability to convert negative feedback into a motivation for “impulse inhibition” (Verdejo-Garcia et al., 2018). When the prefrontal cortex is damaged, the failure of decision-making network integration causes individuals to be dominated by emotions, triggering negative feedback processing disorders and leading to repeated erroneous choices (Bechara et al., 1997). In this study, epileptic patients exhibited reduced sensitivity to negative outcomes and delayed strategy adjustment, which corresponds to the clinical observations of impulsive behaviors and decreased daily decision-making ability in epileptic patients.

This study found that patients with epilepsy showed reduced P300 amplitude and theta oscillation power and lower the percent use of negative feedback compared to the healthy group. Furthermore, the percent use of negative feedback was significantly positively correlated with P300 amplitude. This finding suggests that impaired decision-making of patients with epilepsy prevents them from recognizing errors, processing feedback, or adapting their strategies accordingly. Theta oscillations facilitate coordinated neuronal activity, contributing to P300 generation (Popp et al., 2019). Diminished dopamine levels during decision-making result in reduced activity of the midbrain limbic pathway (Christie and Tata, 2009; van Holst et al., 2010). Lower dopamine levels may be associated with decreased P300 amplitudes and theta oscillations during decision-making in patients with epilepsy. Epilepsy models show reduced hippocampal theta oscillations and poor spatial learning, whereas healthy rats exhibit higher theta coherence during spatial working memory and decision-making tasks (Benchenane et al., 2010). Oscillations support the hippocampus and prefrontal cortex in coordinating activity, enabling efficient information processing and integration. Therefore, P300 and theta oscillations are indicators of compromised decision-making in patients with epilepsy.

Although there was no statistically significant difference between the epilepsy subgroups, the remission group showed better performance than the new-onset epilepsy group in IGT behavioral indicators and P300 amplitude. However, the decision-making capacity of patients with epilepsy in seizure-remission is lower than that of healthy individuals. Electrophysiological data indicate that even after achieving clinical seizure control, patients in seizure-remission still show deficits in error feedback detection and brain oscillatory function. The persistent impact of chronic neural remodeling may alter neuronal connections and functions, impacting decision-making ability even in seizure-remission (Sutula, 2004; Deng et al., 2024).

Clinically, IGT and EEG can be used in combination as cognitive screening tools. Evaluating the decision-making ability of newly diagnosed patients and monitoring P300 amplitude can help early identify individuals at high risk of cognitive decline. For patients in the remission period, early cognitive training combined with cognitive rehabilitation therapy can guide clinical medication adjustment for targeted improvement. This study has certain limitations. First, the sample sizes of the groups were limited and the study did not include multicenter institutional data. Second, The disease course varied between patients with epilepsy in seizure-remission and those with new-onset epilepsy. The course of epilepsy onset is less strongly correlated with decision-making impairments; however, the experimental design of this study could not eliminate the influence of disease course. Third, the influence of medication could not be disregarded in this trial. Therefore, In subsequent studies, we will systematically collect medication-related data for all patients and integrate these variables into statistical analyses to comprehensively investigate the potential impact of medications on research outcomes.

## 5 Conclusion

Patients with GTCS exhibit diminished decision-making capabilities, and those experiencing new-onset epilepsy perform worse, compared with those in seizure-remission. This decline may be associated with a reduced P300 amplitude and diminished theta oscillations. Furthermore, even in the absence of clinical seizures, patients in the seizure-remission phase of epilepsy display some level of decision-making impairment, indicating underlying neurocognitive deficits. This study is constrained by its sample size, and future investigations with large multicenter cohorts are imperative to validate these findings and explore nuanced subgroup differences in decision-making abilities.

## Data Availability

The raw data supporting the conclusions of this article will be made available by the authors, without undue reservation.
